# Intraoperative Dexmedetomidine Improves the Outcome of Pediatric Cardiac Surgery: A One-Year Cohort Study

**DOI:** 10.31083/j.rcm2410289

**Published:** 2023-10-12

**Authors:** Fei Xu, Lei Li, Yanli Yang, Wenjun Liu, Jun Ma, Hushan Ao

**Affiliations:** ^1^Department of Anesthesiology, Beijing Anzhen Hospital, Capital Medical University, 100011 Beijing, China; ^2^Department of Cardiovascular Surgery, Affiliated Hospital of Weifang Medical University, 261053 Weifang, Shandong, China; ^3^Department of Anesthesiology, Cardiovascular Institute and Fuwai Hospital, Chinese Academy of Medical Sciences, 100037 Beijing, China

**Keywords:** dexmedetomidine, pediatric cardiac surgery, outcomes

## Abstract

**Background:**

Pediatric cardiac surgery is associated with a high risk of mortality and morbidity. The aim of this study was to determine if intraoperative dexmedetomidine therapy could improve survival after pediatric cardiac surgery.

**Methods:**

We conducted a retrospective review of 1384 consecutive children who underwent pediatric cardiac surgery. Amongst these, 889 received dexmedetomidine therapy and 495 did not. All children were followed for 1 year. Their in-hospital and long-term outcomes were compared by multivariate logistic regression to minimize bias, and propensity-score matched adjustment was used.

**Results:**

Children who received dexmedetomidine had lower mortality during the 30-day postoperative period compared to children who did not (1.57% vs. 4.24%; adjusted hazard ratio [HR]: 0.448; 95% confidence interval [CI]: 0.219–0.916, *p* = 0.028), as well as after 1 year (2.36% vs. 6.67%; adjusted [HR]: 0.487; 95% [CI]: 0.274–0.867, *p* = 0.014). The two groups showed no significant differences in cardiovascular complications.

**Conclusions:**

Dexmedetomidine administered intraoperatively reduced 30-day and 1-year mortality in children undergoing pediatric cardiac surgery.

## 1. Introduction

Congenital heart disease (CHD) is the leading cause of congenital abnormality at birth, with an estimated worldwide incidence ranging from 4 to 8 per 1000 live births [1]. CHD poses great challenges and is a growing health burden on the quality of life and development of children, their families and society. In recent decades, the incidence of CHD has increased due to environmental pollution and to the increasing number of elderly puerperal [2]. The notable progress in cardiac surgery technology has led to a significant increase in CHD surgery in China to about 40,000 cases per year. The primary therapy to correct CHD is surgical operation performed under general anesthesia. However, infants have poor tolerance to anesthesia due to their light weight, immature development of organs, and imperfect compensatory functions [3]. These factors can lead to serious cardiovascular morbidity and mortality in infancy. The long-term effects of intraoperative anesthesia on important organs and on systemic development are still being hotly debated by clinicians.

Although dexmedetomidine is not approved by the Food and Drug Administration (FDA) for children, it has found a role as an attractive anesthetic adjuvant during CHD surgery in pediatric patients because of its sedative effects and cardiopulmonary profile [4]. Numerous studies have described the clinical effectiveness of dexmedetomidine therapy during congenital heart surgery and in the cardiac intensive care unit (ICU) [5]. Dexmedetomidine has been widely used to simultaneously provide effective sedation, anxiolysis, and to relieve the adverse effects of mechanical ventilation [6]. Moreover, prophylactic and postoperative medication with dexmedetomidine can be used to maintain hemodynamic stability [7] and reduce the intraoperative stress hormone response [8].

There are however potential adverse events from the use of dexmedetomidine for cardiovascular and respiratory effects. The most common clinical presentations of adverse hemodynamic events are bradycardia and hypotension, which occur during the initial loading dose with conduction abnormalities [9, 10]. Nevertheless, these are generally considered to be self-limiting and typically have no clinical significance [11].

A previous study suggested that intraoperative dexmedetomidine could improve survival in adult patients undergoing cardiac surgery [12]. However, adults are physiologically very different to infants, meaning this outcome cannot be extended to infants. So far, no studies have to our knowledge reported the impact of dexmedetomidine on long-term outcomes in pediatric cardiac patients.

In the present study we have retrospectively evaluated the effect of intraoperative dexmedetomidine infusion on outcomes in pediatric cardiac patients.

## 2. Materials and Methods

This single-center, retrospective study examined 1480 consecutive children who underwent pediatric cardiac surgery from January 1st, 2014 to December 31st, 2015.

Children who underwent open-heart surgery for CHD were included in the study. To be eligible, they were required to be younger than 12 months, weigh ≥2.5 kg, and be scheduled for elective cardiac surgery with cardiopulmonary bypass. The exclusion criteria were emergency surgery, significant neurological disorders that prevented accurate titration of sedative and analgesic agents, history of preoperative serious arrhythmia (ventricular fibrillation, third-degree atrioventricular block), or underwent several staging surgeries in one admission. A total of 1384 children were finally included in the study, of which 889 received dexmedetomidine during the intraoperative period and 495 did not. Data was collected for patient characteristics, medical history, preoperative medications, procedural characteristics, and clinical outcomes.

### 2.1 Anesthesia Management

All children were intubated after standard induction with intravenous injection of midazolam, sufentanil and rocuronium bromide. Invasive blood pressure monitoring was performed via the radial or femoral artery. The central venous catheter was usually inserted through the right internal jugular vein. Intraoperative management was left to the discretion of the attending anesthesiologist, with an institutional standard of a moderate dose of midazolam and sufentanil. The decision regarding dexmedetomidine use was solely at the discretion of the attending anesthesiologist. After surgery, all children were transferred to the pediatric intensive care unit (PICU) and managed according to standardized procedures. For children receiving dexmedetomidine, infusion was initiated at 0.2–0.7 ug/kg/h after the central venous catheter was inserted and until extubation in the PICU. The dose of dexmedetomidine was adjusted according to the child’s hemodynamic response.

### 2.2 Study Outcomes

Postoperative complications were reported according to the consensus definitions of the Multi-Societal Database Committee for Pediatric and Congenital Heart Disease. Primary outcomes were 30-day postoperative and 1-year survival rates after surgery. Secondary outcomes included postoperative cardiac arrest, reoperation, acute kidney injury, sepsis, intraoperative urine output, postoperative mechanical ventilation hours, PICU hours, and length of hospital stay.

### 2.3 Statistical Analysis

Continuous variables are presented as mean ± standard deviation (SD) and compared using *t* tests. Categorical variables are shown as frequencies and percentages, and compared using χ^2^ tests. In-hospital outcomes were compared using multiple logistic regression models and multiple linear models adjusted by patient characteristics and operative variables as independent variables. Kaplan-Meier curves and survival analyses were used to determine the effect of dexmedetomidine infusion on survival after pediatric cardiac surgery. Hazard ratios (HRs) were estimated by Cox proportional-hazard regression models. Confounders were included in the models if *p* ≤ 0.05.

Propensity-score matching (PSM) was also performed, whereby 462 children without dexmedetomidine infusion were matched in a 1:1 ratio to those who received dexmedetomidine infusion.

All reported *p*-values are two-tailed and *p* ≤ 0.05 was considered to indicate statistical significance. All statistical analyses were conducted using SAS software (Version 9.4, SAS Institute, Cary, NC, USA).

## 3. Results

### 3.1 Baseline Characteristics

Of the 1384 eligible children, 889 received dexmedetomidine therapy and 495 did not. The two groups had similar baseline characteristics with respect to age, gender, premature infant, pulmonary arterial hypertension, hemoglobin concentration, ALT, AST, ejection fraction and New York Heart Association class (NYHA). However, patients in the dexmedetomidine group had higher weight (6.73 ± 1.63 vs. 6.23 ± 1.88), oxygen saturation (90.8 ± 10.3 vs. 88.5 ± 12.8), albumin (40.8 ± 3.2 vs. 39.8 ± 3.8), but lower creatinine (26.4 ± 7.7 vs. 27.8 ± 10.6) and urea nitrogen (3.05 ± 1.36 vs. 3.21 ± 1.48).

In addition, the dexmedetomidine group had shorter cardiopulmonary bypass time compared with the non-dexmedetomidine group. There were no significant differences between the two groups for cross-clamp time, surgery time and cardiopulmonary bypass (CPB). Following adjustment by PSM, the baseline characteristics between the two groups were balanced (Table 1).

**Table 1.** **Demographic and clinical characteristics**.

Characteristics		Entire cohort		*p* value	Propensity-matched cohort		*p* value
		DEX (N = 889)	Non-DEX (N = 495)		DEX (N = 462)	Non-DEX (N = 462)	
Age (month)		5.93 ± 2.59	5.71 ± 3.22	0.190	5.96 ± 2.74	5.79 ± 3.16	0.566
Weight		6.73 ± 1.63	6.23 ± 1.88	<0.001	6.44 ± 1.63	6.39 ± 1.84	0.547
Gender (Male)		595 (66.93)	319 (64.44)	0.349	307 (66.45)	299 (64.72)	0.631
Premature infant		17 (1.91)	13 (2.63)	0.382	10 (2.16)	11 (2.38)	1.000
Pulmonary arterial hypertension		413 (46.46)	239 (48.28)	0.514	229 (49.57)	230 (49.57)	1.000
Oxygen saturation%		90.8 ± 10.3	88.5 ± 12.8	<0.001	89.2 ± 11.5	89.4 ± 12.0	0.737
Hemoglobin concentration (g/L)		120.4 ± 40.1	120.4 ± 25.6	0.999	120.8 ± 25.2	119.4 ± 25.2	0.389
Creatinine (umol/L)		26.4 ± 7.7	27.8 ± 10.6	0.014	26.5 ± 8.7	27.5 ± 10.3	0.091
Urea nitrogen (mmol/L)		3.05 ± 1.36	3.21 ± 1.48	0.038	3.10 ± 1.44	3.23 ± 1.50	0.151
ALT (IU/L)		30.4 ± 38.8	28.9 ± 21.4	0.443	31.3 ± 50.8	29.0 ± 21.1	0.385
AST (IU/L)		46.6 ± 45.1	45.6 ± 28.1	0.663	49.6 ± 59.0	44.7 ± 21.4	0.402
Albumin (mg/L)		40.8 ± 3.2	39.8 ± 3.8	<0.001	40.1 ± 3.3	40.1 ± 3.6	0.620
Ejection fraction		67.2 ± 6.2	67.5 ± 6.9	0.522	67.6 ± 6.0	67.5 ± 6.8	0.833
NYHA				0.732			0.333
	I	50 (5.62)	29 (5.86)		20 (4.33)	27 (5.84)	
	II	755 (84.93)	413 (83.43)		389 (84.20)	386 (83.55)	
	III	84 (9.45)	53 (10.71)		53 (11.47)	49 (10.61)	
Perfusion time (min)		95.9 ± 49.0	104.5 ± 56.3	0.007	100.6 ± 46.3	101.2 ± 50.8	0.871
Cross clamp time (min)		60.9 ± 32.9	64.8 ± 37.8	0.071	63.7 ± 34.6	63.6 ± 37.4	0.981
Surgery time (min)		180.1 ± 66.5	187.1 ± 82.9	0.109	185.7 ± 70.1	185.6 ± 80.3	0.985

DEX, dexmedetomidine; NYHA, New York Heart Associatio; ALT, alanine amiotransferase; AST, aspartate aminotransferase.

### 3.2 Perioperative Outcomes

Multiple logistic regression analysis showed that dexmedetomidine infusion was not associated with reoperation, sepsis or acute kidney injury (Table 2). However, patients receiving dexmedetomidine had reduced cardiac arrest (0.45% vs. 2.22%, *p* = 0.013). In multiple linear regression analysis, dexmedetomidine infusion was not associated with postoperative mechanical ventilation hours, PICU hours and length of hospital stay (Table 3). However, patients who received dexmedetomidine had more intraoperative urine output (7.16 ± 5.14 vs. 6.14 ± 5.00). PSM analysis confirmed the initial results of reduced incidence of cardiac arrest (0.216% vs. 1.73%, *p* = 0.039) and more intraoperative urine output (7.02 ± 5.45 vs. 6.17 ± 4.47, *p* = 0.009) for patients treated with dexmedetomidine.

**Table 2.** **In-hospital outcomes according to dexmedetomidine status**.

Variable	Entire cohort		Adjusted OR	95% CI	*p* value	Propensity-matched cohort		*p* value
	DEX	Non-DEX				DEX	Non-DEX	
	N = 889	N = 495				N = 462	N = 462	
Cardiac arrest	4 (0.450)	11 (2.22)	0.223	0.068–0.731	0.013	1 (0.216)	8 (1.73)	0.039
Reoperation	9 (1.01)	11 (2.22)	0.569	0.227–1.425	0.229	9 (1.95)	7 (1.52)	0.804
Acute kidney injury	18 (2.02)	22 (4.44)	0.476	0.246–0.920	0.077	10 (2.16)	14 (3.03)	0.523
Sepsis	23 (2.59)	15 (3.03)	1.040	0.518–2.087	0.912	1 (0.216)	5 (1.08)	0.219

DEX, dexmedetomidine; OR, odds ratio; CI, confidence interval.

**Table 3.** **In-hospital outcomes according to dexmedetomidine status**.

Variable	Entire cohort		B	95% CI	*p* value	Propensity-matched cohort		*p* value
	DEX	Non-DEX				DEX	Non-DEX	
	N = 889	N = 495				N = 462	N = 462	
Intraoperative urine output (mL/Kg/h)	7.16 ± 5.14	6.14 ± 5.00	1.064	0.525–1.603	<0.001	7.02 ± 5.45	6.17 ± 4.47	0.009
Postoperative mechanical ventilation hours	61.3 ± 176.3	64.8 ± 141.4	–0.233	–18.108–17.641	0.980	69.2 ± 183.0	55.2 ± 111.9	0.147
PICU hours	121.4 ± 257.3	126.7 ± 182.8	6.721	–18.533–31.976	0.602	125.5 ± 209.2	116.1 ± 161.6	0.431
Length of hospital stay (days)	11.8 ± 9.7	12.0 ± 8.5	0.507	–0.489–1.505	0.318	12.1 ± 9.3	11.6 ± 7.6	0.279

DEX, dexmedetomidine; PICU, pediatric intensive care unit; CI, confidence interval.

### 3.3 Survival Outcomes

**Fig. 1.**
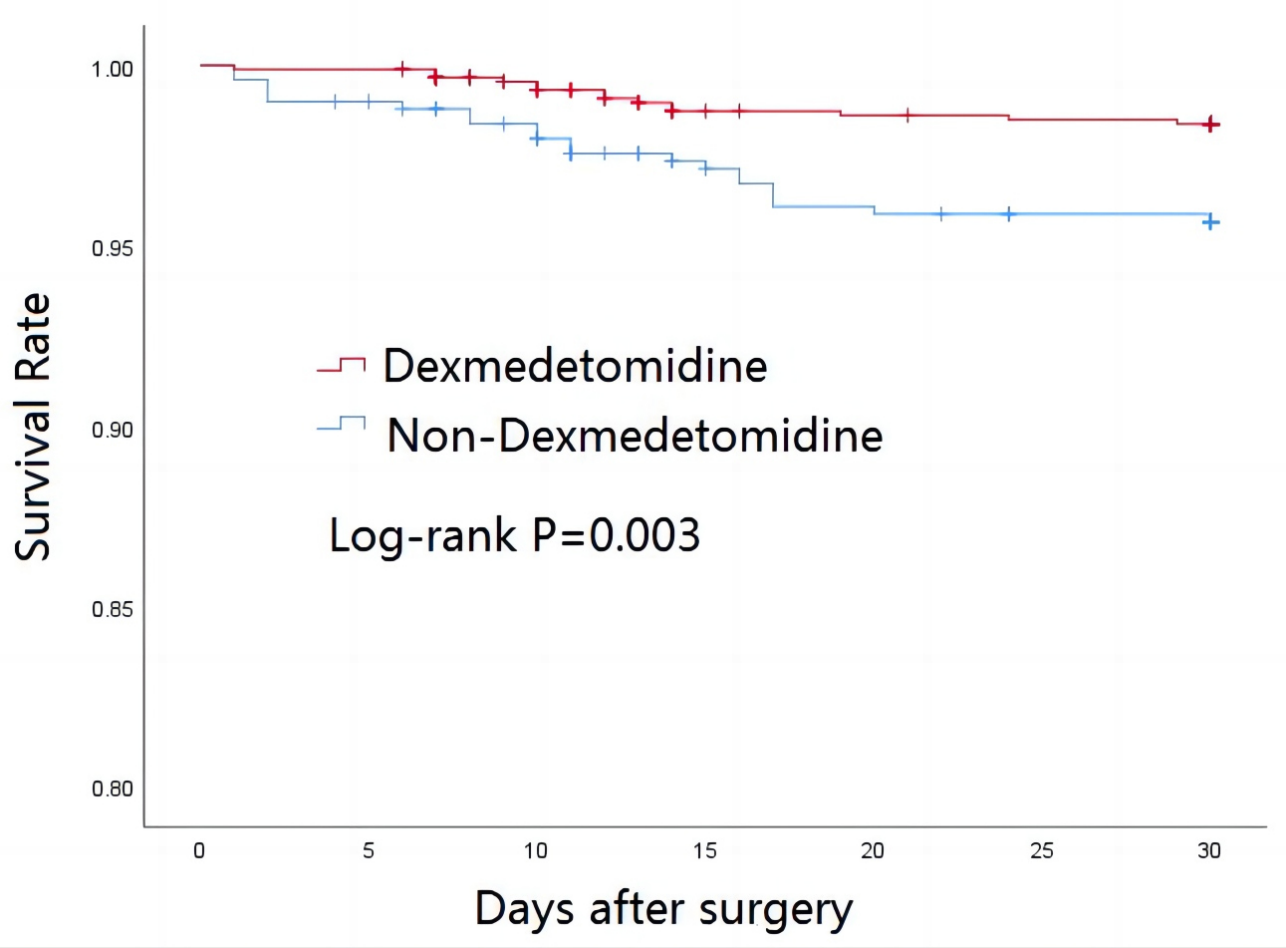
**Thirty-day survival rate according to dexmedetomidine status**.

**Fig. 2.**
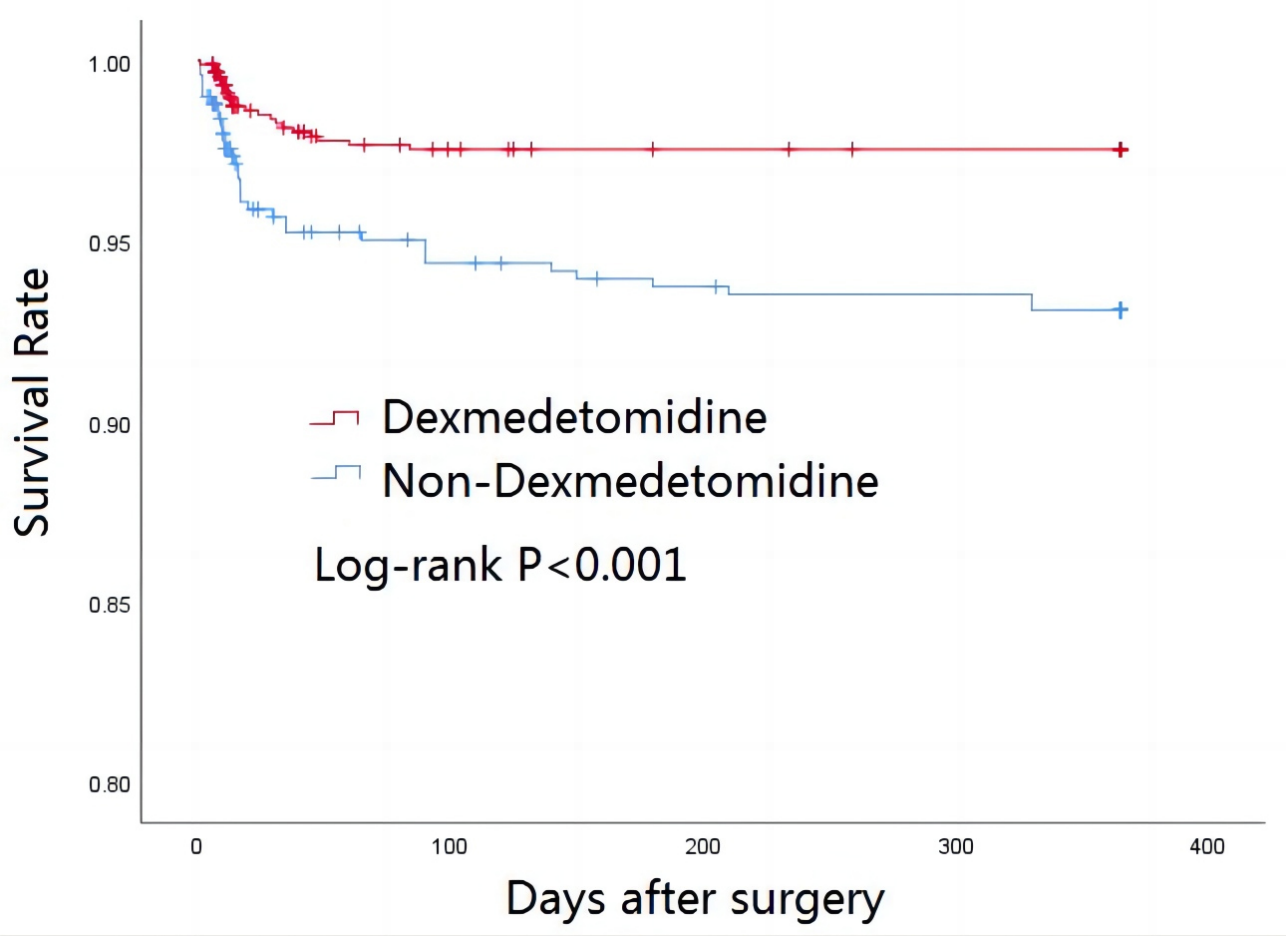
**One-year survival rate according to dexmedetomidine status**.

After 30 days of follow-up, 35 (2.53%) patients had died of various causes, while 54 (3.90%) patients had died after 1-year. Cox proportional hazard analysis showed that patients with dexmedetomidine infusion had better 30-day postoperative survival (adjusted HR, 0.448; 95% CI: 0.219–0.916, *p* = 0.028) and 1-year postoperative survival (adjusted HR, 0.487; 95% CI: 0.274–0.867, *p* = 0.014). Kaplan-Meier curves for 30 day and 1-year postoperative survival are shown in Figs. 1,2, respectively. Log-rank tests found significant differences in survival between the two groups for both time periods. Following PSM, the differences between the groups in 30-day mortality (1.51% vs. 3.90%, *p* = 0.027) and in 1-year mortality (2.81% vs. 5.63%, *p* = 0.031) remained significant (Table 4).

**Table 4.** **Long-term outcomes according to dexmedetomidine status**.

Variable	Entire cohort		Adjusted HR	95% CI	*p* value	Propensity-matched cohort		*p* value
	DEX	Non-DEX				DEX	Non-DEX	
	N = 889	N = 495				N = 462	N = 462	
30-day mortality	14 (1.57)	21 (4.24)	0.448	0.219–0.916	0.028	7 (1.51)	18 (3.90)	0.027
1-year mortality	21 (2.36)	33 (6.67)	0.487	0.274–0.867	0.014	13 (2.81)	26 (5.63)	0.031

DEX, dexmedetomidine; HR, hazard ratio; CI, confidence interval.

## 4. Discussion

To our knowledge, this is the first study to evaluate the long-term safety and efficacy of dexmedetomidine for children undergoing pediatric cardiac surgery. We found that intraoperative dexmedetomidine infusion was associated with lower 30-day (1.57% vs. 4.24%) and 1-year (2.36% vs. 6.67%) postoperative mortality in consecutive cases of children undergoing pediatric cardiac surgery. Our results also suggest that intraoperative dexmedetomidine infusion can reduce the incidence of cardiac arrest after surgery, and significantly improve intraoperative urine output.

Approximately 30–50% of infant mortality is due to CHD, despite significant improvements in surgery, anesthesia and ICU management [13]. Many more patients also suffer complications such as arrhythmia, neurologic dysfunction, renal injury, prolonged mechanical ventilation, and reoperation.

Although dexmedetomidine does not have FDA approval for use in pediatric patients, it is used with increasing frequency as an anesthetic adjuvant in children undergoing surgery for CHD [8]. Dexmedetomidine has sedative effects without causing significant respiratory depression, thus alleviating the use of opioids and benzodiazepines. This pharmacological profile can lead to better patient-ventilator interaction and less delirium [6, 14]. In addition, experimental studies have shown that dexmedetomidine has organ protective effects [15]. The clinical benefits of dexmedetomidine in adults are widely recognized. Previous studies in adult patients undergoing cardiac surgery have shown that intraoperative dexmedetomidine infusion was associated with improved 1-year postoperative survival and less overall postoperative complications [12]. Our earlier study found that intraoperative dexmedetomidine administration could reduce postoperative atrial fibrillation in adults [16]. However, adults are physiologically different to children with CHD, and hence these findings cannot be extrapolated to children. So far, no studies have to our knowledge reported the impact of intraoperative dexmedetomidine infusion on long-term outcomes following pediatric cardiac surgery, especially in infants.

In the largest study to date, analysis of a CHD database by the congenital cardiac anesthesia society-society of thoracic surgeons (CCAS-STS) found that dexmedetomidine was preferentially used in older or larger infants undergoing less complex CHD surgery [17]. However, the conclusion that dexmedetomidine improved outcomes in children with CHD could not be drawn, since no adjustment was made for confounding variables. In the present study, patients who underwent CHD surgery were younger than 12 months. Our results showed that intraoperative dexmedetomidine was associated with reduced short- (30 days) and long-term (1-year) mortality rates in infants undergoing CHD surgery.

This study also found that dexmedetomidine provided significant benefits against cardiac arrest. In animal studies, post-conditioning with dexmedetomidine improved post-resuscitation cardiac and neurological outcomes in a dose-dependent manner by inhibiting tissue inflammation, oxidative stress, cell apoptosis and necroptosis [18]. Studies have also shown that dexmedetomidine has a protective effect on the myocardium. α_2_-adrenergic agonists are known to have a protective effect on myocardial ischemia by increasing cAMP levels and enhancing adenosine-induced coronary vasodilatation. Preconditioning with dexmedetomidine has been shown to attenuate myocardial ischemia/reperfusion injury by activating pro-survival kinases [19, 20]. Both preconditioning and postconditioning with dexmedetomidine can therefore effectively protect the heart and brain from ischemia-reperfusion injury [21, 22].

In agreement with a previous report [23], the current study found that intraoperative urine output was higher during dexmedetomidine infusion. Experimental animal studies have shown that dexmedetomidine may induce free-water diuresis and micturition by inhibiting neuronal signaling in large cells of the paraventricular nucleus in the hypothalamus [24]. Dexmedetomidine can also decrease the level of a kidney injury marker [25]. These actions may protect the kidneys during ischemic events.

Several statistical methods were used to adjust for patient characteristics and clinical risk factors. Multiple logistic-regression analysis and Cox regression analysis were used to provide comprehensive comparisons of the treatment groups. In addition, the PSM approach was used to estimate the two derived groups, and all baseline covariates between the two groups were balanced.

This study has some limitations. First, it was a retrospective and non-randomized study performed at a single center. Second, many dates were abandoned in PSM analysis because the two groups were unmatched. Third, although PSM analyses were carried out to reduce selection bias between two groups, potential flaws may still exist in this nonrandomised study. Every patient did receive dexmedetomidine in the post-operative period, we just analysis intraoperative dexmedetomidine to the outcomes. Further high-quality, multicenter randomized controlled trial (RCT) are required to confirm the beneficial effects of dexmedetomidine in pediatric patients undergoing heart surgery for CHD.

## 5. Conclusions

Intraoperative administration of dexmedetomidine reduces short- and long-term mortality in children undergoing pediatric cardiac surgery.

## Data Availability

The datasets used and/or analyzed during the current study are available from the corresponding author on reasonable request.
